# Targeting the proteasome subunit PSMB5 by RNA interference induces proteasome dysfunction and mortality in the Colorado potato beetle (*Leptinotarsa decemlineata*)

**DOI:** 10.1038/s41598-025-28793-x

**Published:** 2025-11-21

**Authors:** Leonie Graser, Eric RL Gordon, Matthew Jamison, Win Talton, Yuting Chen, Eileen Knorr, Anton Windfelder, Kenneth Narva, Andreas Vilcinskas

**Affiliations:** 1 Institute for Insect Biotechnology, Justus Liebig University, 35390 Giessen, Germany; 2 Branch Bioresources, Fraunhofer Institute for Molecular Biology and Applied Ecology IME, 35392 Giessen, Germany; 3 GreenLight Biosciences, Research Triangle Park, NC 27709 USA; 4 Department of Diagnostic and Interventional Radiology (Experimental Radiology), University Hospital Giessen, Giessen, Germany

**Keywords:** RNA interference, Functional genomics, Pest control, Proteomics, Ubiquitin, Biotechnology, Molecular biology, Plant sciences

## Abstract

The Colorado potato beetle (CPB, *Leptinotarsa decemlineata*) is a devastating super-pest of potato crops in the northern hemisphere. Chemical control methods are expensive, harmful to non-target species, and inefficient because CPB populations can rapidly evolve resistance to multiple classes of insecticides. Double-stranded RNA (dsRNA) provides an opportunity for the sustainable and targeted control of CPB by RNA interference (RNAi). A dsRNA active ingredient (ledprona, marketed as Calantha^®^) targeting proteasome subunit β5 (PSMB5) achieves the efficient suppression of PSMB5 gene expression and protein synthesis, causing CPB mortality and protecting crop plants in the laboratory and field. To understand the mode of action of ledprona in more detail, expression levels of other proteasome subunit genes and proteins and correspondingly the accumulation of ubiquitinated proteins were measured in ledprona-treated CPB larvae. This study confirms the strong downregulation of PSMB5 mRNA and PSMB5 protein, as well as the systemic upregulation of other proteasome transcripts and the accumulation of cell waste in the form of ubiquitin-labeled degrons that correlate with larval mortality. This comprehensive characterization of ledprona’s mode of action expands our previous knowledge of the only sprayable dsRNA pesticide with regulatory registration in the United States and may facilitate future efforts to control CPB.

**Supplementary Information:**

The online version contains supplementary material available at 10.1038/s41598-025-28793-x.

## Introduction

The Colorado potato beetle (CPB, *Leptinotarsa decemlineata*) is a devastating pest of potato crops (*Solanum tuberosum*) and has a global distribution exceeding 16 million km^2^^1^. Crop damage is caused throughout the CPB life cycle, from early-instar larvae to adults, and amounts to billions of USD in losses^2,3^. Adults undergo an overwintering diapause phase and can mate soon after emergence, with females laying 300–800 eggs each^4–6^. The high fecundity and aggressive foraging and feeding behavior of CPB can lead to rapid population growth and distribution^7,8^. CPB has also evolved to efficiently detoxify and tolerate glycoalkaloids, which are toxic insecticidal compounds produced by its solanaceous host plants. The high reproductive potential and predisposition for resistance has contributed to the emergence of CPB populations that are unaffected by many of the chemical insecticides currently on the market^9–11^. This increases the pressure to identify new pest management solutions for the effective and sustainable control of CPB^2^.

RNA interference (RNAi) is a promising pest control method with a species-specific mode of action^12–15^. RNAi can be triggered by double-stranded RNA (dsRNA), which is taken up into cells and cleaved into small interfering RNA (siRNA) by the endonuclease Dicer-2, which is associated with dsRNA-binding protein R2D2^12,16–18^. The 21-bp siRNA then binds to Argonaute 2, which separates the strands and forms an RNA-induced silencing complex (RISC) with the guide strand, enabling it to form base pairs with complementary mRNA^19^. This results in the cleavage of the target mRNA and the suppression of protein synthesis^20^. In agricultural settings, RNAi can be triggered by the ingestion of dsRNA that has been sprayed onto host plants, known as spray-induced gene silencing (SIGS), or expressed in host plants, known as host-induced gene silencing (HIGS)^21^. Given the target specificity of RNAi and its low environmental impact, sprayable RNA-based biopesticides are favored for the development of integrated pest management systems^22,23^.

**Fig. 1:**
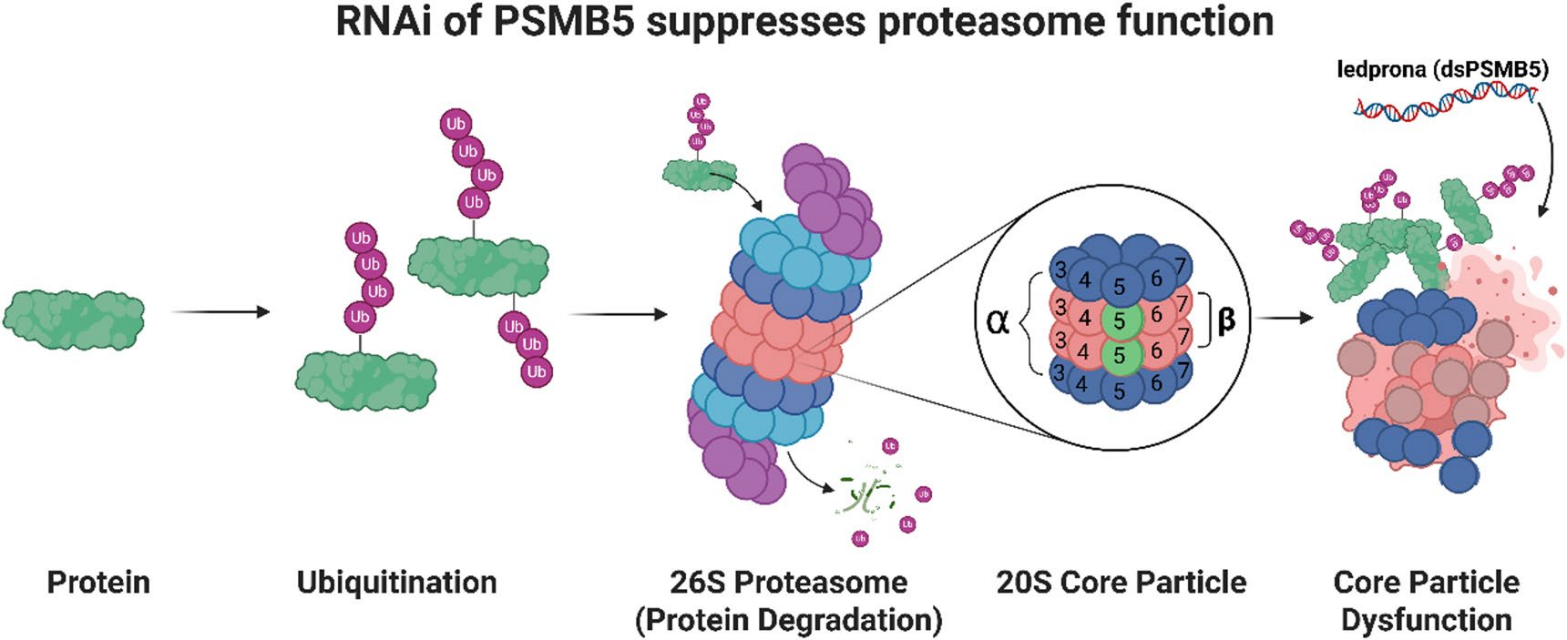
The activity of *dsPSMB5* (ledprona) and how it affects proteasome function. Proteins are labeled with ubiquitin chains (four or more ubiquitin units) and are brought to the proteasome for degradation. The *PSMB5* gene (encoding the two β5 subunits of the core particle, marked in green) is suppressed by *dsPSMB5*. This results in a dysfunctional core particle that is unable to process proteins, leading to the lethal accumulation of unprocessed ubiquitinated proteins. Created in BioRender. G, L. (2025) https://BioRender.com/rpi9t4y.

A SIGS dsRNA (*dsPSMB5*) targeting the essential β5 subunit (PSMB5) of the 26 S proteasome core particle has been registered by the US Environmental Protection Agency (EPA) and marketed as Calantha^®^ for the control of CPB^24,25^. The 26 S proteasome is a specialized proteolytic complex found in many eukaryotic cells and is responsible for the recycling of damaged and misfolded proteins that impair important cellular functions if allowed to accumulate^26,27^. The 26 S proteasome is composed of two 19 S activator regulatory particles that recognize and deliver proteins labeled for degradation, and one 20 S proteolytic core particle where most protein degradation occurs^28^. The core particle contains four homologous seven-ring cylindrical structures comprising multiple α and β subunits, including PSMB5. The proteasome complex is part of the ubiquitin–proteasome degradation system, in which conserved 76 -residue ubiquitin units are attached to selected proteins by a system of enzymes to signal proteolysis in the proteasome^26,27,29^. Ubiquitin is recruited by the E1 ubiquitin-activating enzyme, E2 ubiquitin-conjugating enzyme, and specialized E3 enzyme, which together form a ubiquitin-ligase complex that ensures specific binding to the protein target^29,30^. The covalent attachment of four or more polymerized ubiquitin molecules results in the delivery of tagged proteins to the proteasome complex, where they are digested and recycled in the 20 S core particle^26,31^. Ledprona targets *PSMB5* mRNA and thus prevents the synthesis of β5 subunits that are essential for the functionality of the 20 S core particle **(**Fig. 1**)**. The prevention of proteolysis is assumed to cause the buildup of cell waste and ultimately cell death.

Here we investigated the detailed mode of action of ledprona by examining the molecular basis of proteasome dysfunction. We confirmed the direct knockdown of *PSMB5* mRNA and PSMB5 protein but expanded our analysis to include all α and β subunits of the 20 S proteasome core particle. We also investigated the ubiquitin pooling dynamics in ledprona-treated cells and the correlation between larval mortality and the accumulation of unprocessed ubiquitin-tagged proteins.

## Results

### Impact of Ledprona on larval survival

**Fig. 2:**
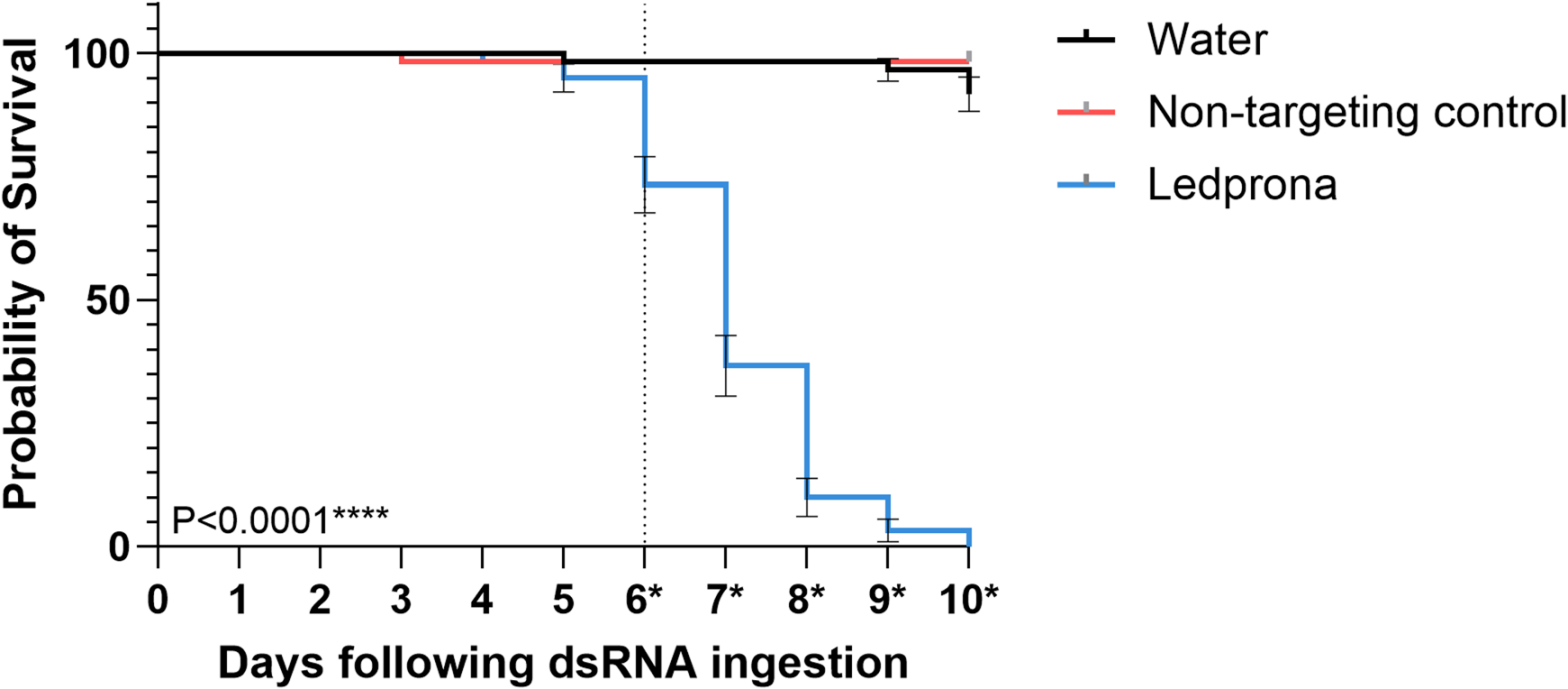
Survival curves of L2 larvae fed on potato leaf discs treated with 0.2 g/L ledprona (*dsPSMB5*), 0.2 g/L of the non-target control (*dsGFP*) or water (*n* = 60) for 10 days. Ledprona-treated CPB larvae demonstrated a significantly lower probability of survival than the control groups. Survival was plotted using Kaplan–Meier statistics and significance was determined using the log-rank Mantel–Cox test (****p < 0.0001). Days where significance begins are marked with asterisks (*). Error bars denote standard errors of the mean.

The analysis of L2 larvae exposed to 50 µL of ledprona at a concentration of 0.2 g/L (1 × 10^− 5^ g total RNA per leaf) revealed a significant (*p* < 0.0001) increase in mortality starting 6 days after treatment compared to both the non-targeting dsRNA control (0.2 g/L *dsGFP*) and water as a negative control **(**Fig. 2**)**. Sixty individuals were observed per group with three replicates of 20 larvae each. After 10 days, mortality rates for the water and *dsGFP* controls were only 8% and 3%, respectively. In contrast, ledprona-treated larvae showed limited feeding behavior after only 3 days (data not shown), with mortality first observed on day 5. Mortality became significant (63%) after 6 days, and increased to 90% after 8 days, 96% after 9 days, and 100% after 10 days.

### Comprehensive molecular analysis of Ledprona activity at the mRNA and protein levels

**Fig. 3:**
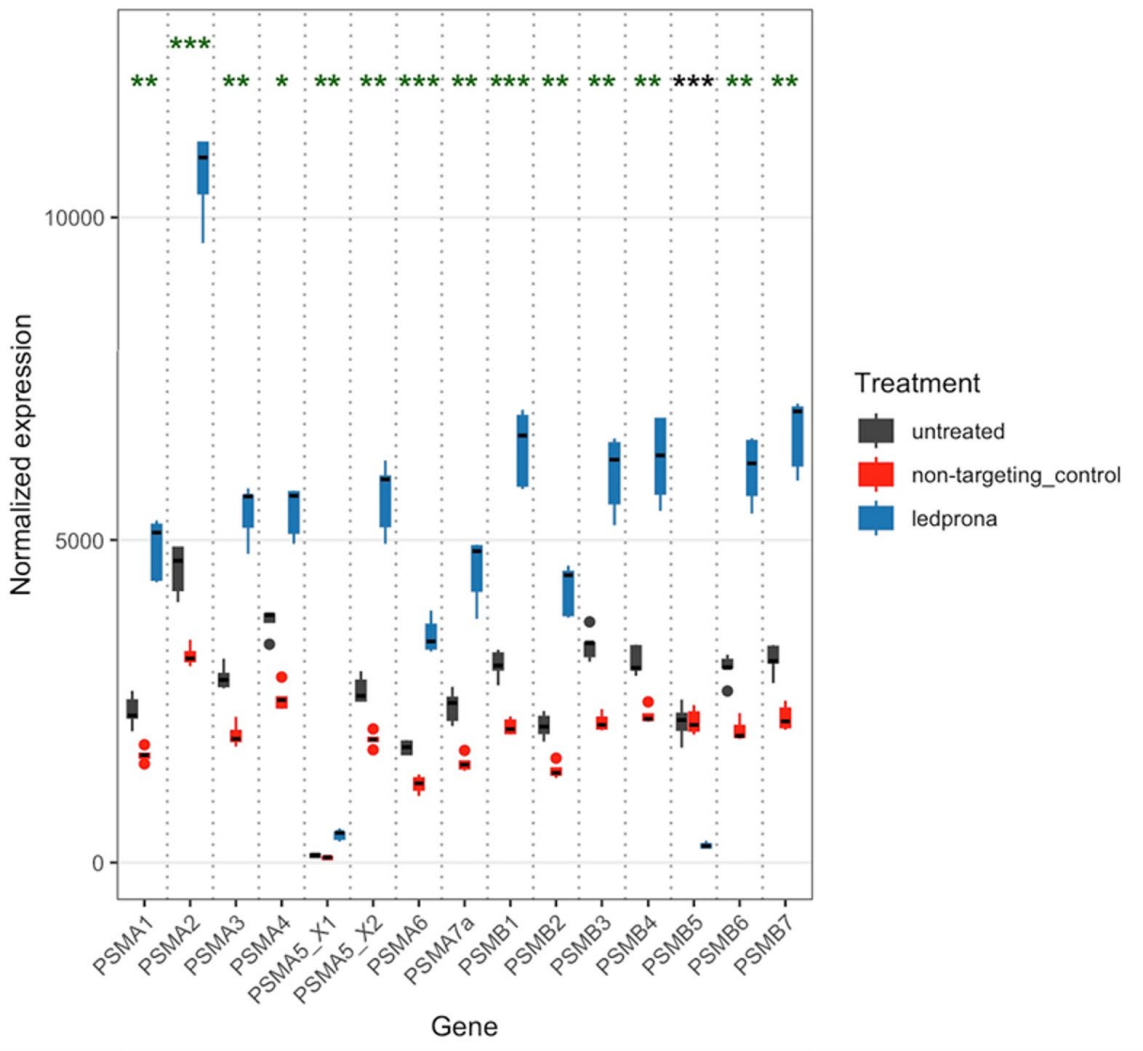
Normalized expression of proteasome core subunits 72 h after treatment with ledprona (*dsPSMB5*), nonspecific control dsRNA (*dsGFP*) or water. A Wald test for significance was implemented in DeSeq2. Asterisks represent adjusted p-values (*0.05–10^− 5^, **10^− 5^–10^− 20^, ***< 10^− 20^). Green and black asterisks show genes upregulated and downregulated, respectively, in the ledprona group compared to controls.

We recovered between 28 and 36 million sequencing reads per sample and our raw data is deposited at the NCBI Sequence Read Archive (SRA). A BUSCO score of 94.8% ensured that the reference genomic resource we used to map our RNA-seq data was of high quality. Transcripts representing the core subunits of the barrel structure of the proteasome were quantified 72 h after treatment with ledprona, *dsGFP* or water **(**Fig. 3**)**. *PSMB5* mRNA was significantly downregulated in the CPB larvae fed ledprona-treated leaf discs compared to both negative controls, whereas mRNA encoding all other α and β subunits, non-core subunits of the proteasome, and other proteasome-related proteins were upregulated (Fig. 3; Figure S1; Table S1-S2). In the ledprona-treated larvae, *PSMB5* mRNA levels were 88% and 86% lower compared to the *dsGFP* and water controls, respectively (Fig. 3). We found no evidence of sequencing reads derived directly from the dsRNA, which would have confounded our gene expression calculations (Figure S2). We also observed the significant upregulation of certain genes included in a panel of 115 RNAi-related transcripts, with some upregulated only in ledprona-treated larvae compared to non-target controls (Figure S3; Table S1 & S3). Notably, the *Dicer-2* and *Ago2* genes were upregulated in the presence of both ledprona and *dsGFP* (Figure S3; Table S3). The most significantly upregulated genes in the ledprona-treated larvae compared to non-target controls encoded the chaperone proteins DNAJA1, CRYAB and L(2)EFL, and the proteasome-associated proteins ADRM1 and PSMC4 **(**Table 1**)**. The most significantly downregulated gene in the ledprona-treated larvae was *PSMB5* as anticipated (Table 1; Figure S4; Table S2 & S4).

**Table 1:** Statistical and expression data for the top five most significantly upregulated and downregulated genes in ledprona-treated RNA-Seq libraries compared to non-target controls.

	Gene	Full gene annotation	Transcript accession	Adjusted *p*-value	Fold Change
**Upregulated**	DNAJA1	dnaJ homolog subfamily A member 1 (LOC111517751)	XM_023174013.1	7.36E-145	3.03
	CRYAB	α-crystallin B chain-like (LOC111505783)	XM_023160649.1	8.09E-140	98.26
	L(2)EFL	protein lethal(2)essential for life-like (LOC111505782)	XM_023160648.1	8.44E-131	92.25
	ADRM1	proteasomal ubiquitin receptor ADRM1-like (LOC111509886)	XM_023165742.1	9.06E-129	2.36
	PSMC4	26 S proteasome regulatory subunit 6B (LOC111507999)	XM_023163409.1	1.69E-128	3.04
**Downregulated**	PSMB5	proteasome subunit β5 (LOC111503877)	XM_023158308.1	4.88E-132	0.12
	PGLP1	phosphoglycolate phosphatase 1B, chloroplastic-like (LOC111512465)	XM_023168586.1	3.17E-62	0.44
	HPGD	15-hydroxyprostaglandin dehydrogenase [NAD(+)]-like (LOC111509361)	XM_023165086.1	3.88E-30	0.15
		uncharacterized LOC111509358 (LOC111509358)	XM_023165080.1	3.27E-25	0.1
	RALDH1	retinal dehydrogenase 1-like (LOC111506486)	XM_023161556.1	3.75E-23	0.52

### Absolute quantification of PSMB5 peptide levels

**Fig. 4:**
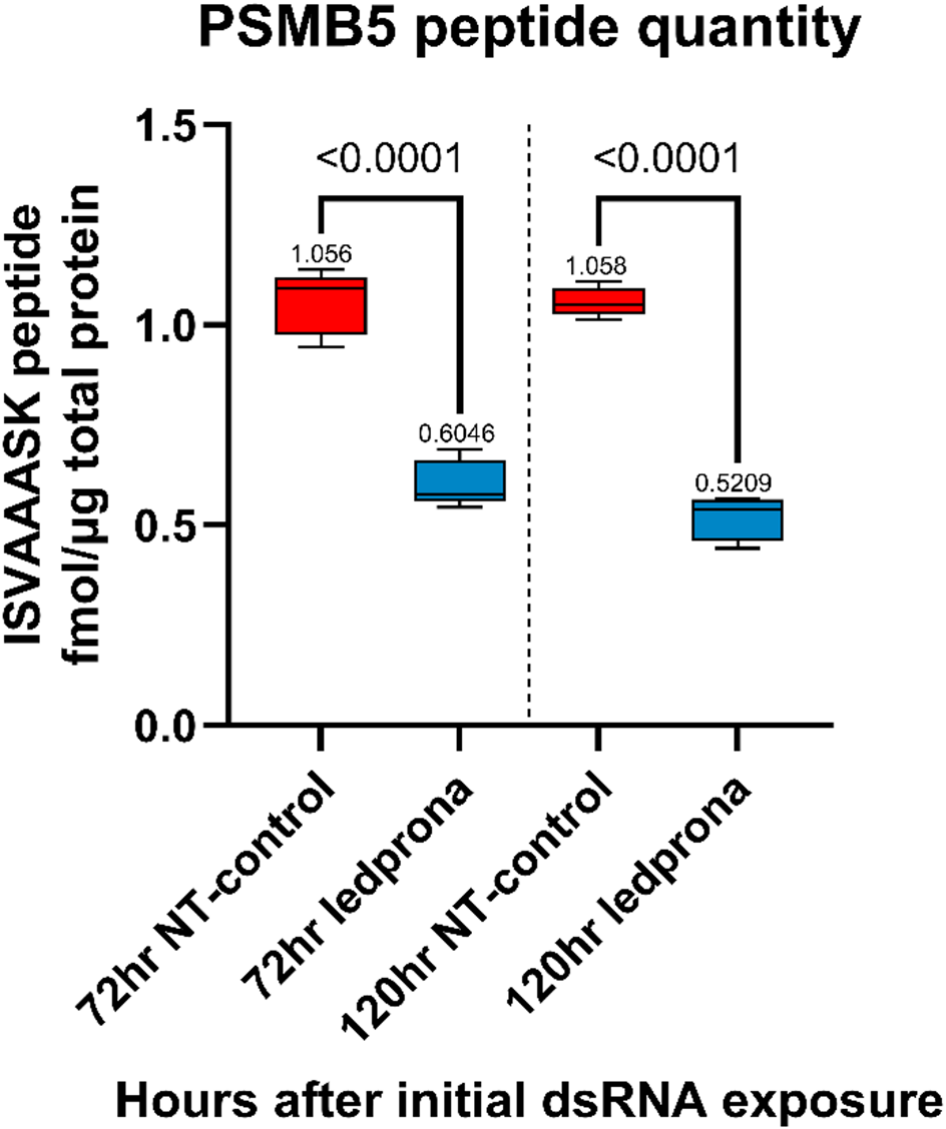
Absolute quantification of the PSMB5 peptide 72 and 120 h after treatment with ledprona (*dsPSMB5*) or the non-target control (*dsGFP*). Data are means of five replicate treatments each involving five pooled larvae (*n* = 25). Statistical significance was determined using an unpaired t-test with Welch’s correction.

Absolute quantitation of the PSMB5 peptide following larval exposure to ledprona or *dsGFP* confirmed its significant (*p* < 0.0001) depletion in the ledprona-treated larvae after 72 and 120 h compared to the *dsGFP* control **(**Fig. 4**)**. The quantity of PSMB5 peptide in the ledprona-treated larvae was 43% lower than in the control after 72 h and 51% lower after 120 h. There was no significant difference (*p* > 0.05) between the two time points in the *dsGFP* control. Our proteomics data therefore supported our RNA-Seq data, confirming the statistically significant downregulation of *PSMB5* mRNA and PSMB5 peptide levels as early as 72 h post-exposure.

### Normalized expression of proteasome β subunit peptides after Ledprona exposure

**Fig. 5:**
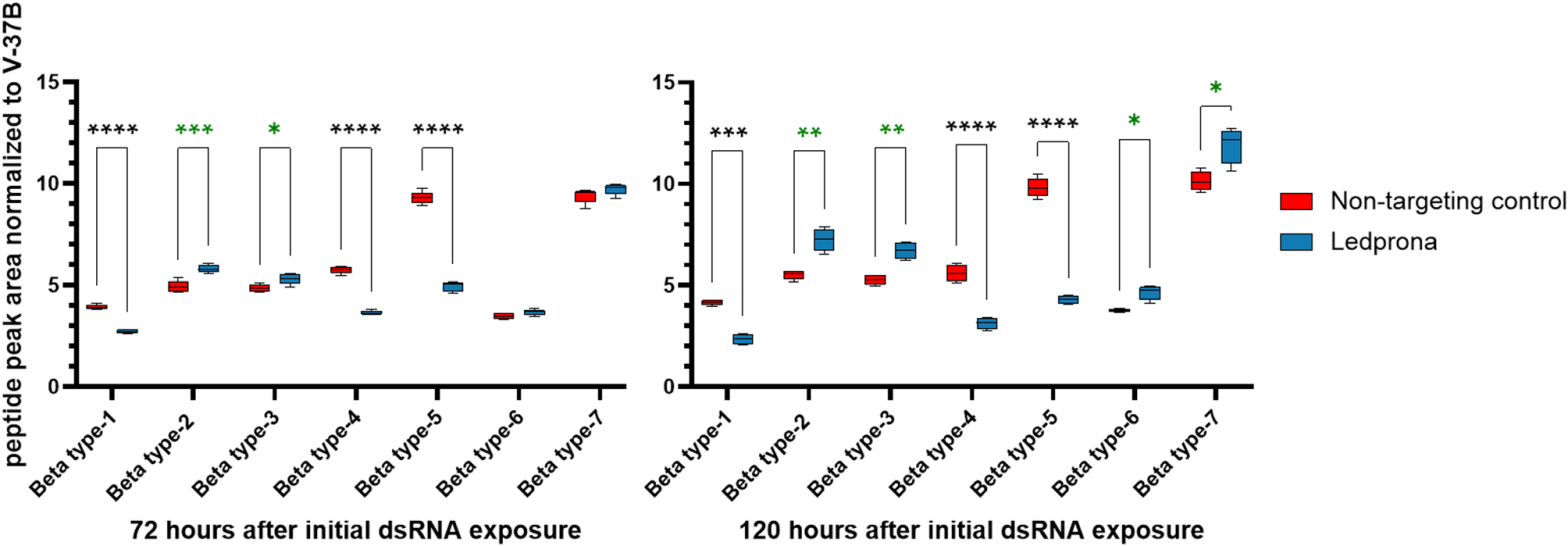
Normalized expression levels of proteasome β-type subunit peptides 72 and 120 h after treatment with ledprona (*dsPSMB5*) or control (*dsGFP*). Data are means of five replicate treatments each involving five pooled larvae (*n* = 25). Statistical significance was determined using a multiple unpaired t-test with FDR adjustment. Results are marked in green if peptides are significantly more abundant in the ledprona libraries, and in black if significantly less abundant. The data for subunit β7 at 72 h did not pass the normality test and a Mann-Whitney U-test was applied (*0.01 < *p* < 0.05, **p < 0.01, ***p < 0.001, ****p < 0.0001).

The abundance of β subunit peptides was determined 72 and 120 h after exposure to ledprona or *dsGFP* and each value was normalized against the housekeeping protein V-37B, which was selected because the corresponding mRNA showed low variance in the RNA-Seq dataset (Figure S5). We observed significant depletion (*P* < 0.0001) of the β1, β4 and β5 subunits in the ledprona-treated larvae at both time points **(**Fig. 5**)**. The PSMB1, PSMB4 and PSMB5 peptides showed 0.69-fold, 0.64-fold and 0.53-fold changes in abundance, respectively, relative to the *dsGFP* control after 72 h, and 0.57-fold, 0.56-fold and 0.44-fold changes, respectively, after 120 h. In contrast, we observed a significant increase in the abundance of the β2, β3, β6 and β7 subunits. The PSMB2 peptide was 1.18-fold more abundant after 72 h and 1.32-fold more abundant after 120 h, compared to the *dsGFP* control. Similarly, the PSMB3 peptide was 1.1-fold more abundant after 72 h and 1.27-fold more abundant after 120 h. The PSMB6 and PSMB7 peptides only became more abundant after 120 h, with increases of 1.24-fold and 1.18-fold, respectively, compared to the *dsGFP* control. These data mostly correlate with the RNA-Seq results showing the significant upregulation of all genes encoding α and β subunits except PSMB5. However, subunits β1, β4 and β5 were clearly downregulated at the protein level in contrast to the upregulation of the corresponding transcripts.

### Absolute quantification of ubiquitin peptides

**Fig. 6:**
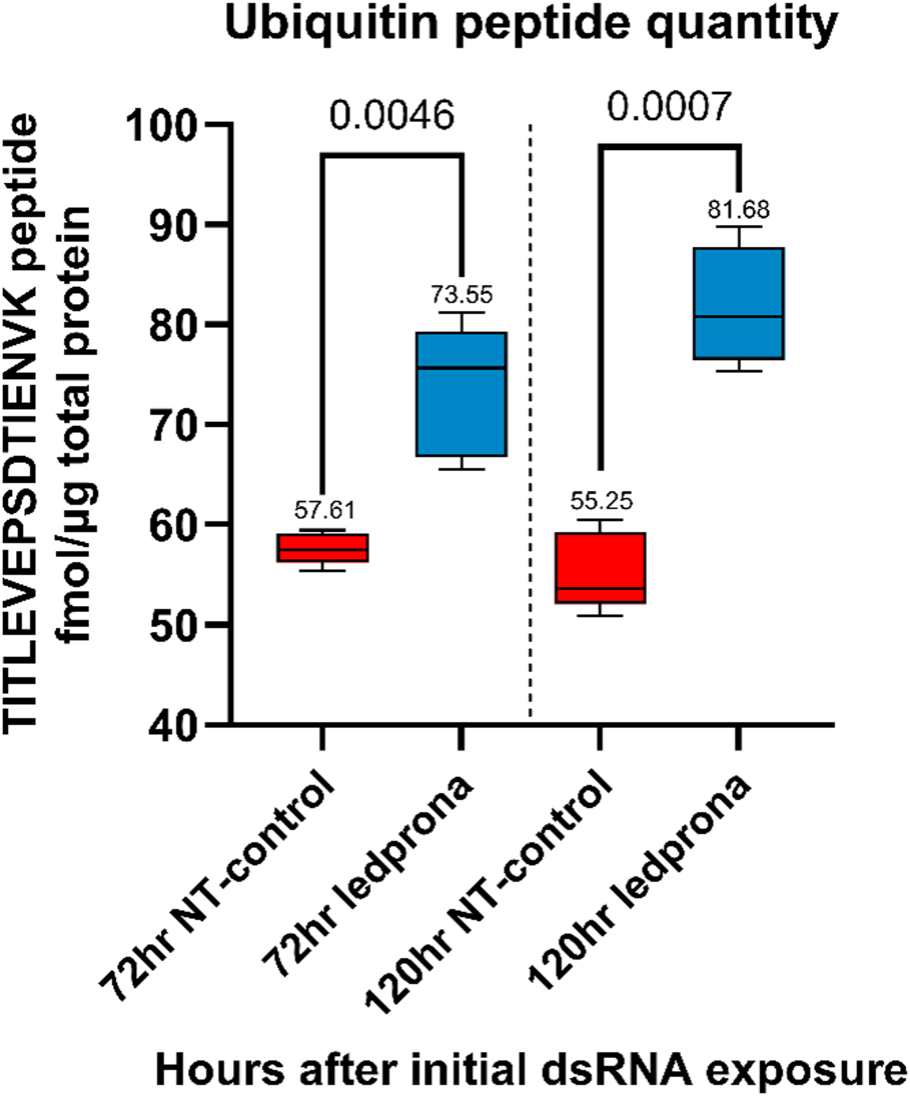
Absolute quantification of ubiquitin peptides 72 and 120 h after treatment with ledprona (*dsPSMB5*) or *dsGFP*. Data are means of five replicate treatments each involving five pooled larvae (*n* = 25). Statistical significance was determined using an unpaired t-test with Welch’s correction.

To determine whether the function of the 20 S core proteasome particle was disrupted by ledprona treatment, resulting in the accumulation of unprocessed ubiquitinated proteins, we measured the absolute level of ubiquitin peptides. We observed a statistically significant (*P* < 0.005) 1.28-fold increase in the accumulation of ubiquitin peptides 72 h after ledprona treatment compared to the *dsGFP* control, becoming an even more significant (*p* < 0.001) 1.48-fold increase after 120 h **(**Fig. 6**)**. There was no significant difference between the time points in the *dsGFP* control (*p* > 0.05). The accumulation of ubiquitin peptides as early as 72 h after ledprona treatment supports the RNA-Seq and peptide quantification data showing the progressive depletion of *PSMB5* mRNA and PSMB5 protein.

## Discussion

CPB is a devastating agricultural pest primarily due to its fecundity and ability to adapt rapidly to a wide range of chemical insecticides^1,9,32^. This adaptive capacity underscores the need for sustainable pest management solutions with novel modes of action. Ledprona (*dsPSMB5*), a 490-bp sprayable dsRNA targeting an essential subunit of the proteasome core particle, is the active ingredient of the biopesticide Calantha^®^ (IRAC group 35) that utilizes RNAi to effectively control CPB^25,33^.

With novel mechanisms of action, there is always a risk of resistance development with improper incorporation into grower IRM (Insecticide resistance management) programs. While the exact mechanisms of resistance to dsRNA are not yet well understood in CPB, studies are trying to understand how resistance develops in Coleoptera. In the western corn rootworm (WCR; *Diabrotica virgifera virgifera)*, a colony resistant to the transgenic crop expressing DvSnf7 dsRNA has been used as an important tool to understand dsRNA resistance in insects and develop effective IRM strategies for dsRNA products^34,35^. The resistance appeared to be autosomal and recessive, suggesting the rate of resistance can be decreased with proper mitigation strategies, such as refuge planting^34^.Refuge planting maintains genetic diversity in pest populations by ensuring sufficient numbers of susceptible insects, which promotes mating between resistant and susceptible individuals that result in heterozygous offspring sensitive to insecticidal proteins and dsRNA. Additionally, the characterization of the first-dsRNA resistant insect population from WCR demonstrated impaired luminal uptake and resistance that was not dsRNA specific^34^. Clathrin-mediated endocytosis (CME) and SID-like (SIL) proteins are critical for the uptake of dsRNA in multiple insect species, including CPB^36–38^ In the mosquito, *Aedes aegypti*, a reduction of dsRNA uptake following the inhibition of the CME pathway was circumnavigated through the use of “paperclip” dsRNA structures^38^. Paperclip dsRNA have closed ends which can continue inducing RNAi in cells where the CME pathway is inhibited, suggesting there are other Clathrin-independent mechanisms which remain susceptible^38^. The proper integration and mitigation strategies are essential for incorporation of insecticidal dsRNA products to ensure that resistance is slowed and in best cases avoided. Calantha^®^ is a non-transgenic, sprayable form of dsRNA, the first product listed in the IRAC group 35 classification, recognizing the product as a novel mechanism of action. Calantha^®^ can be effectively integrated into grower programs in rotation with other insecticides for mitigation against the development of CPB resistance^39^. Additionally, there is low likelihood of cross resistance to chemical insecticides, and other biological insecticides such as *Bacillus thuringienis* (Bt), as laboratory-generated strains of CPB resistant to dsRNA remained susceptible to conventional insecticides^34,40^ The successful management of CPB through the use of sprayable dsRNA suggests that other coleopteran pests could be similarly managed by inducing RNAi.

Following the ingestion of ledprona, significant mortality was observed within 5–6 days (Fig. 2) and limited feeding behavior was noted as early as day 3. The significant downregulation of *PSMB5* mRNA and PSMB5 peptide levels was observed 72 and 120 h after exposure to ledprona, but not in larvae fed on the nontargeting control *dsGFP* (Figs. 3, 4 and 5; Table 1). Interestingly, the genes encoding all other α and β subunits of the 20 S core particle were upregulated in the ledprona-treated larvae but not in the *dsGFP* control, suggesting this is a compensatory response to the depletion of PSMB5 and not a general consequence of RNAi. When also comparing ledprona libraries to the non-target control samples, the most significantly upregulated genes encoded the chaperones DNAJA1, CRYAB and L(2)EFL and additional proteasome-associated proteins ADRM1 and PSMC4 (Table 1). As anticipated, the most significantly downregulated gene was PSMB5, but others encoded the enzymes phosphoglycolate phosphatase 1B, 15-hydroxyprostaglandin dehydrogenase and retinal dehydrogenase (Table 1; Figure S4). A global characterization of gene expression changes due to RNAi via RNA-seq is not available in many systems, but up and downregulation of non-target genes has been observed before at least in *Tribolium castaneum*^41^ and *Varroa destructor* (Smeele at al., in prep). Although in our study all untargeted α and β subunit genes were upregulated by ledprona treatment, the levels of PSMB1 and PSMB4 peptides were lower after exposure, perhaps due to the lack of overall proteasome structural integrity (Fig. 5). PSMB1, PSMB2 and PSMB5 are composed of proteolytically active centers, which are activated during the late stages of proteasome assembly and are responsible for the recruitment and incorporation of other propeptides^42,43^. The β5 propeptide is specifically responsible for interactions with UMP1, an assembly chaperone for the proteasome. In yeast, deletion of the β5/Pre2 propeptide was lethal because it inhibited the assembly of the core particle^43^. Furthermore, β subunits contain C-terminal tails that are essential for proteasome biogenesis and control the specific interaction within or between β rings^43^. The lack of these essential subunit building blocks may therefore cause the proteasome to lose structural integrity and functionality. Additionally, the core particle of the proteasome in eukaryotic cells is assembled initially via an α ring-like structure that acts as the backbone for β subunit assembly^44^. The β subunit precursors assemble in a specific order and are cleaved either immediately before or during the dimerization of the two α/β-ring complexes^44^. PSMB5 depletion may therefore prevent the dimerization of the two halves of the 20 S core particle, resulting in proteasomal dysfunction.

We confirmed that the depletion of PSMB5 leads to protein aggregation caused by the accumulation of ubiquitin-tagged proteins (Fig. 6). We also provided evidence that the cell responds to the loss of PSMB5 by inducing the expression of genes encoding other essential proteasome subunits, but that only some of the corresponding subunits become more abundant whereas others are less abundant (Fig. 5). This suggests that some form of restricted proteasome functionality affects the survival of CPB larvae, and indicates that targeting other proteasome subunits could be a promising strategy^25,45^. In studies with the red flour beetle *Tribolium castaneum*, a compensatory response involving secondary cysteine peptidases at the mRNA and protein levels was also observed following the knockdown of the primary cysteine peptidase, TC01101^46^. Other studies have combined RNA-Seq and proteomic analysis to investigate proteasome-related gene targets including Prosβ1 in the cabbage stem flea beetle (*Psylliodes chrysocephala*), revealing the depletion of specific transcripts and proteins associated with gene expression and translation, but the secondary effects of ubiquitinated proteins accumulating due to proteasome dysfunction were not investigated^47^.

The recent registration of ledprona (product name Calantha^®^) by the EPA is a significant milestone because it is the first sprayable dsRNA pesticides approved for commercial use^25,48^. This registration underscores its potential as a more sustainable alternative to conventional insecticides, with less risk to non-target species and minimal environmental impact^49,50^. Recent studies with Calantha^®^ demonstrated no evidence of effects on non-target arthropods including beneficial insects, non-pests and other coleopteran species and comparable control of CPB to other available pesticides^33,51^. Our study provides further insights into ledprona’s mode of action, which not only involves the direct suppression of *PSMB5* mRNA and PSMB5 protein synthesis, but also alters the abundance of transcripts and proteins representing the other proteasome subunits, accompanied by the accumulation of ubiquitinylated proteins. This functional characterization of the proteasome when critical subunits of the core particle are depleted provides insights into the ubiquitin–proteasome degradation system and the effects of its disruption on CPB mortality.

## Conclusion

We show how ledprona dsRNA, a registered product for the control of Colorado potato beetle, causes lethal cellular effects in larvae by disrupting the function of proteasomes, triggering the accumulation of dysfunctional and damaged proteins. We provide evidence for the specific knockdown of the target gene (PSMB5) at the mRNA and protein level as well as changes in the expression of other proteasome subunits and the accumulation of ubiquitinated proteins, offering insights into ledprona’s precise mechanism of action.

## Methods

### Ledprona sequence

Ledprona is a 490-bp dsRNA (GenBank accession: XM_023158308.1) flanked by 15-bp internal transcribed spacers (Greenlight Biosciences, Durham, North Carolina, USA). The dsRNA matches the *PSMB5* mRNA sequence in CPB and was produced in a cell-free system (U.S. Patent No. 10,858,385)^25^.

### CPB rearing

The colony was established from beetles collected from fields surrounding Ghent University (Ghent, Belgium) in 2019. Adult beetles were reared at the Fraunhofer IME (Giessen, Germany) in large cages (50 × 50 × 100 cm) filled with a 1:3 mixture of sand and loam and provided with potted potato plants. The cages were placed in a greenhouse at 24 °C with 70% relative humidity and a 16-h photoperiod. The cages were replenished with fresh potato plants twice weekly and eggs were collected every 24 h. The eggs were kept in identical greenhouse conditions and stored in plastic boxes lined with filter paper and supplied with potato leaves, which were refreshed daily until fourth-instar larvae (L4+) were large enough to pupate. Second-instar larvae (L2) were used for all experiments.

### Laboratory leaf disc feeding assay

Potato leaf discs were prepared using a 40-mm hollow punch (Matador, Remscheid, Germany). The discs were then treated with 0.2 g/L dsRNA (ledprona, or *dsGFP* as a nonspecific control), or with water as a negative control, using a dip method which coated both sides of the leaf disc with 50 µL of the applied liquid. The ledprona concentration of 0.2 g/L was selected because this was previously shown to induce 60% mortality by day 5 and 100% mortality by day 8^25^. The coated and air-dried leaf discs were placed in 47-mm Petri dishes (Thermo Fisher Scientific, Schwerte, Germany) lined with filter paper, which was moistened with 150 µL of distilled water. A single L2 larva was placed on each disc using a paintbrush (*n* = 20). The dishes were replenished with freshly treated leaf discs on day 4 and then with untreated leaf material until the end of the assay. The dishes were incubated at 24 °C and 60% relative humidity in a cabinet (Regineering, Preith, Germany). Mortality was recorded every 24 h for 10 days and the assay was carried out three times (*n* = 60). Survival analysis was conducted using the Kaplan–Meier method and treatments were compared using the log-rank Mantel–Cox test^52^.

### Larval sample Preparation

*PSMB5* mRNA and PSMB5 protein expression were measured in homogenates of L2 larvae after 72 and 120 h. Each sample consisted of five pooled larvae with five replicates (*n* = 25). Larvae were collected and immediately frozen in liquid nitrogen before storage at − 80 °C.

### RNA-Seq

RNA-Seq analysis was carried out as previously described^53^. Briefly, total RNA was extracted from larval samples homogenized in 500 µl TRIzol Reagent (Thermo Fisher Scientific). Libraries were constructed using the NEBNex Ultra II Directional RNA Library Prep Kit for Illumina (New England Biolabs, Ipswich, MA, USA) according to the recommended protocol. Library quality was assessed on an Agilent 4200 TapeStation. Paired-end Illumina reads were trimmed to remove adaptor sequences and low-quality bases using Trimmomatic v0.39. Paired forward and reverse reads were mapped to the *Leptinotarsa decemlineata* reference transcriptome (GCF_000500325.1) using hisat2^54^ and salmon^55^ was used to generate metrics for each transcript. DESeq2^56^ was used for differential expression analysis, filtering genes with fewer than 10 reads per transcript and present in fewer than three libraries. The libraries were evaluated for any reads derived directly from the dsRNA sequence by mapping exclusively to the non-target control, ledprona and the target gene sequence using bbmap^57^. A Wald test of significance was implemented in DeSeq2 to determine the significance of upregulation and downregulation. Normalized data was compared to the housekeeping protein V-37B because the corresponding mRNA showed low variance in the RNA-Seq dataset (Figure S5).

### Proteomics

The proteomics analysis was conducted using a targeted approach focusing on the proteasome subunits. Peptides were chosen based on uniqueness, ensuring no other matching peptides existed in the proteasome, and chromatography, meaning the peptides could be detected with the column and solvent gradient used. Total protein was extracted from larval samples using the solvent precipitation SP3 (SP4) method^58^. We then digested 200 µg of protein per sample with trypsin-LysC. Peptides were quantified using a Vanquish HPLC system and a QExactive mass spectrometer with a HESI source. Peptide retention times, normalized collision energies, and transitions for quantification are provided in Table S5 and Table S6. Vascular sorting-associated protein 37B (V-37B) was used as the housekeeping sequence for normalization. The peptides used to monitor the expression of the β subunits were optimized for peak areas (Table S6). Peptide ISVAAASK was used for PSMB5 and peptide TITLEVEPSDTIENVK was used for ubiquitin. An unpaired t-test with a Welch’s correction was used to compare absolute PSMB5 and ubiquitin peptide levels. A multiple unpaired t-test was used with an additional false discovery rate (FDR) adjustment to compare the normalized peptide expression data of the proteasome β subunits. Normalized data was compared to the housekeeping protein V-37B because the corresponding mRNA showed low variance in the RNA-Seq dataset (Figure S5).

## Supplementary Information

Below is the link to the electronic supplementary material.

Supplementary Material 1

Supplementary Material 2

Supplementary Material 3

Supplementary Material 4

Supplementary Material 5

## Data Availability

The datasets generated and/or analyzed during the current study are available in the Sequence Read Archive (SRA) repository at the following link: [https://www.ncbi.nlm.nih.gov/sra/PRJNA1299307].
