# Supplementary material for: Targeting the proteasome subunit PSMB5 by RNA interference induces proteasome dysfunction and mortality in the Colorado potato beetle (Leptinotarsa decemlineata)
Source: Sci Rep. 2025 Nov 21;15:41183. doi: 10.1038/s41598-025-28793-x (PMC12639069; doi:10.1038/s41598-025-28793-x)
Supplement: Supplementary file 1 — Supplementary Material 1 [file 41598_2025_28793_MOESM1_ESM.pdf]

Supporting Information

Targeting the Proteasome Subunit PSMB5 by RNA Interference Induces Proteasome Dysfunction and Mortality in the Colorado Potato Beetle (*Leptinotarsa decemlineata*)

Leonie Graser[1,2], Eric Gordon[3], Matthew Jamison[3], Win Talton[3], Yuting Chen[3], Eileen Knorr[2], Anton Windfelder[2,4], Kenneth Narva[3], Andreas Vilcinskas[1,2]**

L. Graser, A. Vilcinskas
[1] Institute for Insect Biotechnology, Justus Liebig University, Giessen, 35390, Germany

L. Graser, E. Knorr, A. Windfelder, A. Vilcinskas
[2] Branch Bioresources, Fraunhofer Institute for Molecular Biology and Applied Ecology IME, Giessen, 35392, Germany

E. Gordon, M. Jamison, W. Talton, Y. Chen, K. Narva
[3] GreenLight Biosciences, Research Triangle Park, NC, 27709, United States

A. Windfelder
[4] Department of Diagnostic and Interventional Radiology (Experimental Radiology), University Hospital Giessen, Giessen, Germany

*Leonie Graser, Ohlebergsweg 12, 35392, Giessen, Germany, +49 641-972-19248

*Andreas Vilcinskas, Ohlebergsweg 12, 35392, Giessen, Germany, +49 641-972-19100

Email: leonie.graser@uni-giessen.de;andreas.vilcinskas@ime.fraunhofer.de

This PDF file includes:

Supporting text

Figures S1 to S5

Tables S5 to S6

Methods

RNA-Seq

Total RNA was checked for quality on an Agilent 4200 TapeStation System. Total RNA concentrations were determined using a NanoDrop 8000 spectrophotometer. The mRNA was isolated from 1 μg of total RNA using the NEBNext Poly (A) mRNA Magnetic Isolation Module (NEB). Library concentrations were determined using a Qubit 2.0 fluorimeter (Thermo Fisher Scientific) with the Qubit dsDNA BR Assay Kit (Thermo Fisher Scientific). Library concentrations were then normalized and pooled in equal amounts. The final library concentration was 750 pM with 5% PhiX. The libraries were run on an Illumina NextSeq 1000 with a P2 100-cycle cartridge to produce 800 million 50-bp paired-end reads.

Proteomics

The Vanquish system was optimized for μLC using nanoViper tubing at the following positions: injector to column (75 μm × 350 mm), column to divert valve (75 μm × 750 mm), and divert valve to source (75 μm × 350 mm). The column for peptide separation was a Phenomenex Kinetex C18, 2.1 × 150 mm, 1.7 μm particle size. We used solvent A (Milli-Q water with 0.1% formic acid) and solvent B (acetonitrile with 0.1% formic acid) and the gradient method conditions are shown in Table S1. Peptide data were collected using a parallel reaction monitoring method with an injection volume of 5 μL, a resolution of 35,000, an AGC target of $1×10^5$, a maximum IT of 200 ms, and an isolation window of 2.0 kDa.

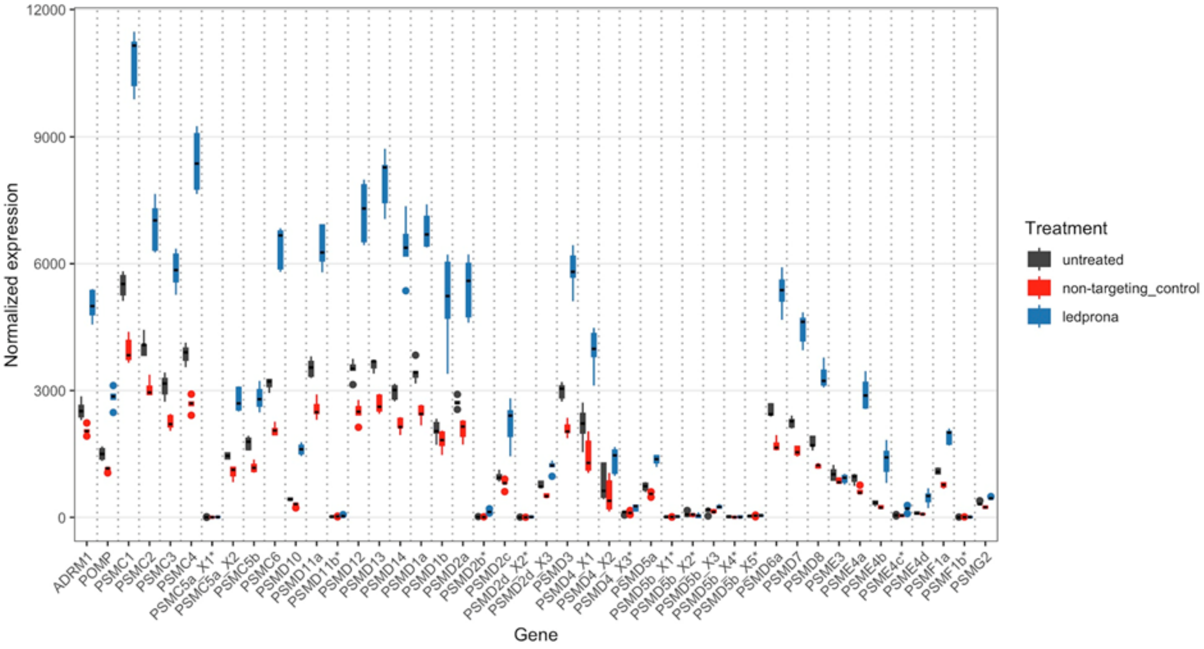

Fig. S1. Normalized expression of 44 non-core subunits of the proteasome and other associated proteins for RNA-Seq libraries 72 h after treatment with ledprona (*dsPSMB5*), *dsGFP* or water.

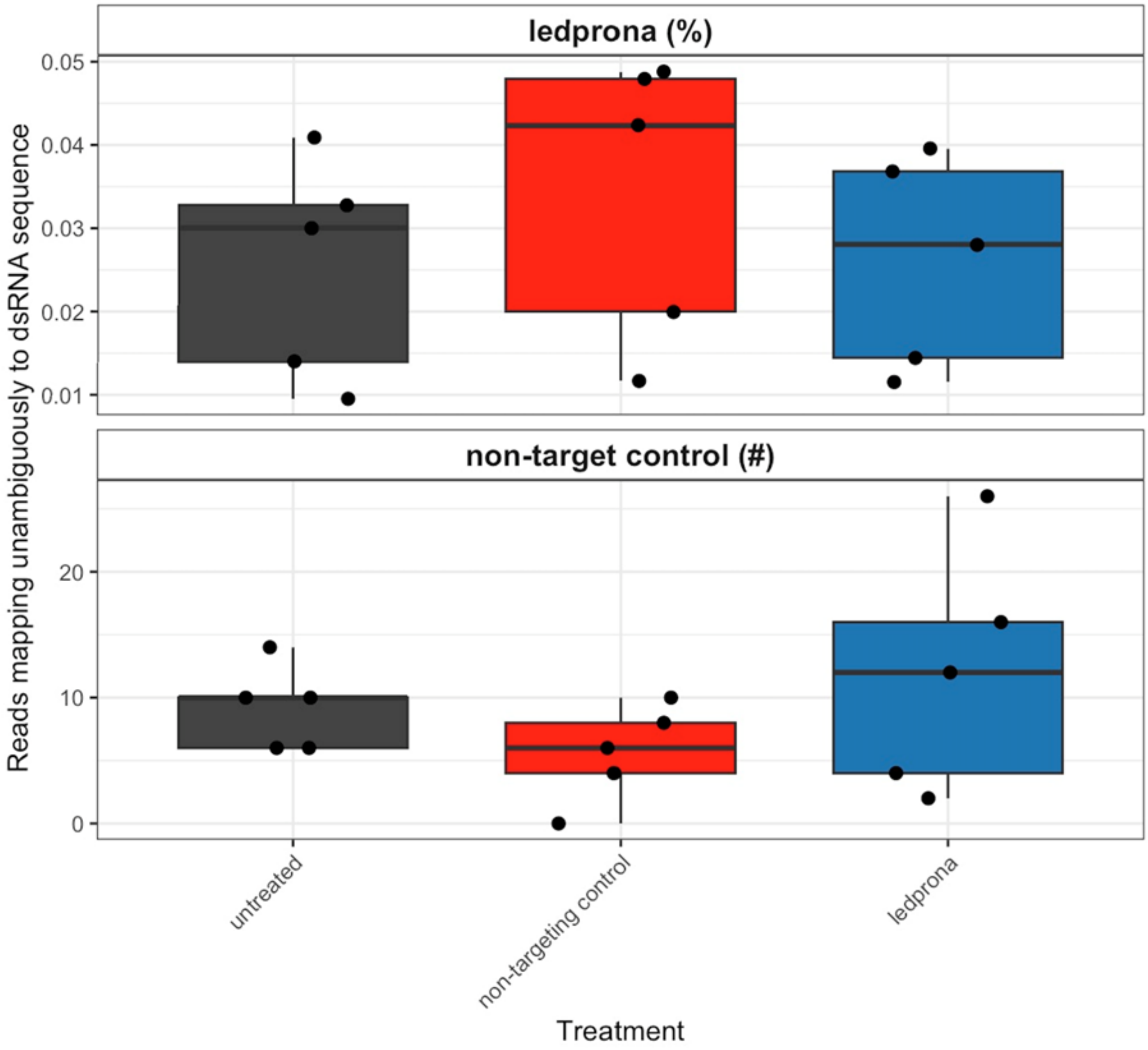

Fig. S2. RNA-Seq reads mapping exclusively to the dsRNA treatment sequence. For the ledprona sequence (top), percentages of ledprona-mapping reads compared to the total number of reads mapping to either the mRNA of the target gene (*PSMB5*) or ledprona are plotted. For *dsGFP* (bottom), the total number of reads is plotted.

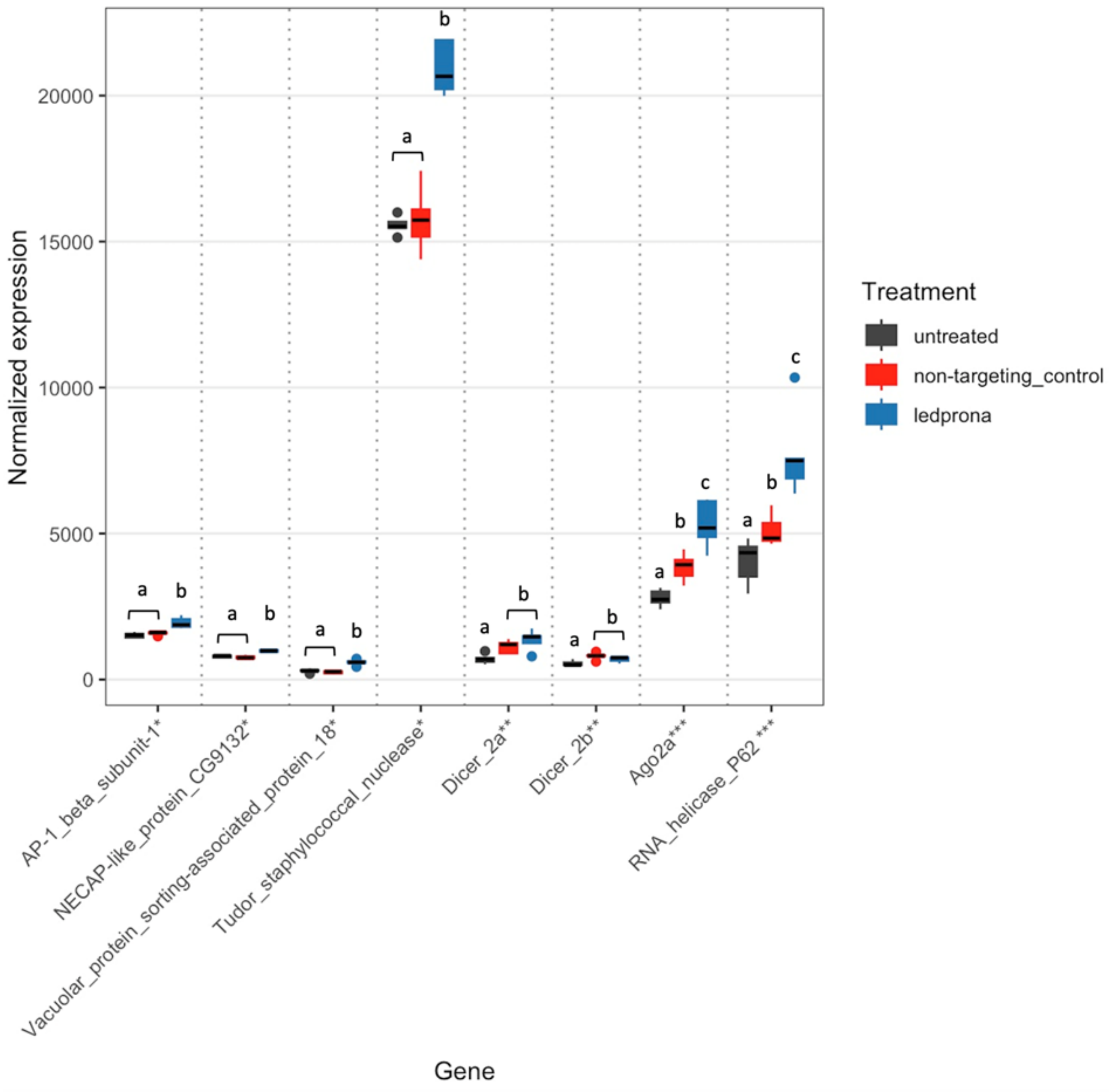

Fig. S3. Normalized expression of RNAi-associated genes with significant upregulation in ledprona treated RNA-Seq libraries 72 h after treatment with ledprona (*dsPSMB5*), *dsGFP* or water. Results from a Wald test of significance implemented in DeSeq2 are shown for genes upregulated in response to any dsRNA in addition to genes upregulated in ledprona-treated RNA-Seq libraries.

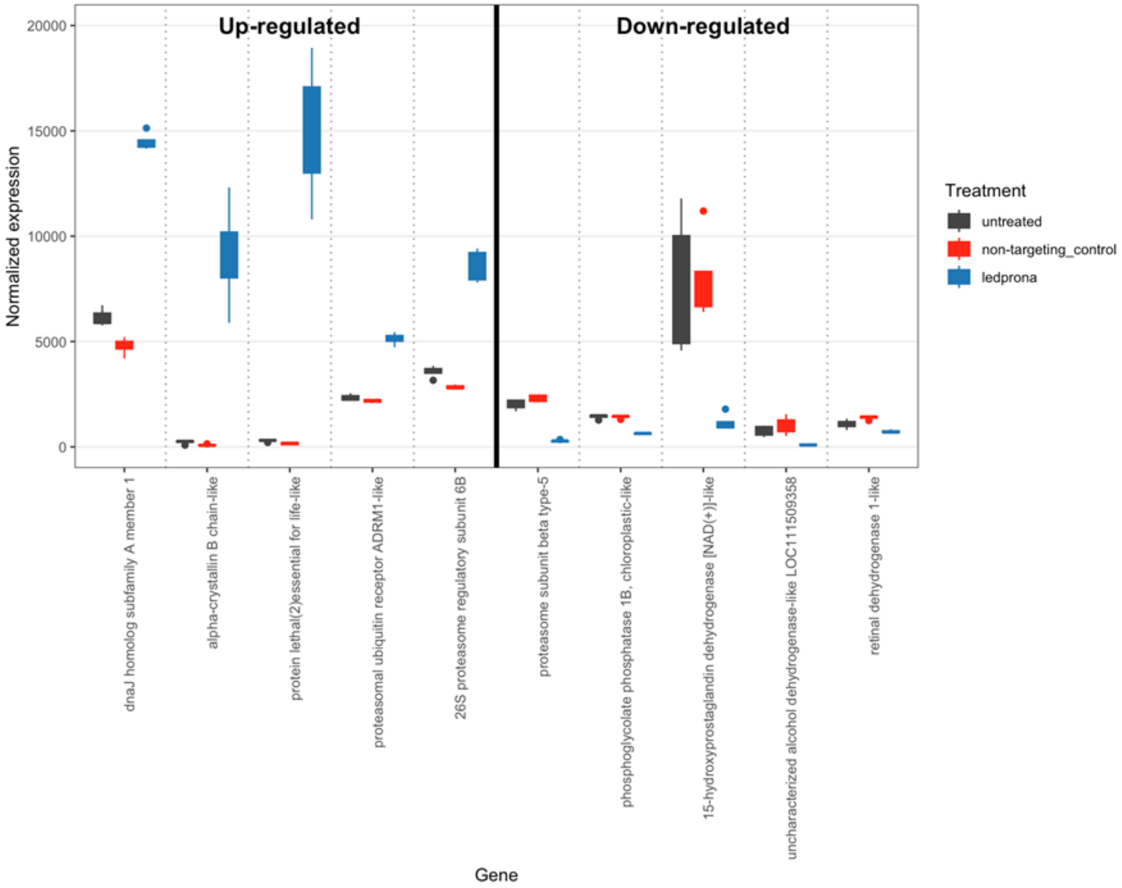

Fig. S4. Normalized expression of the top five most significantly upregulated and downregulated genes in ledprona-treated RNA-Seq libraries compared to non-target *dsGFP* controls.

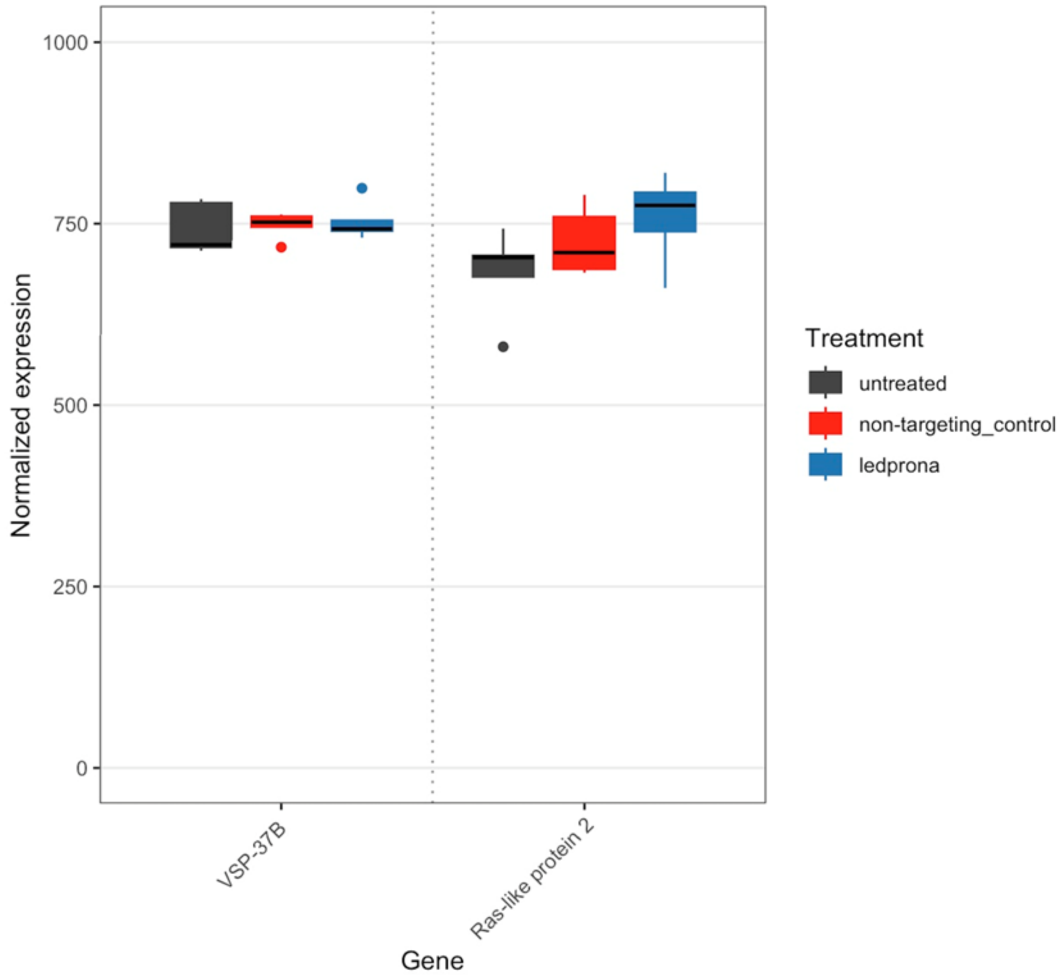

Fig. S5. Normalized expression of two stable genes – vacuolar protein sorting-associated protein 37B (*VSP-37B*) and Ras-like protein 2 (*R-Ras2*). VSP-37B was selected for the normalization of proteomic samples for RNA-Seq libraries 72 h after treatment with ledprona (dsPSMB5), dsGFP or water.

Table S5. HPLC gradient method (%B refers to the proportion of solvent B in the gradient elution profile; Gradient curve references the solvent gradient and the rate it changes (5 is a standard linear gradient used to quantify the target proteins))

Time (min)	Flow (mL/min)	%B	Gradient curve
0	0.15	3	5 = linear
0.5	0.15	3	5 = linear
60	0.15	35	5 = linear
60.01	0.15	95	5 = linear
63	0.15	95	5 = linear
63.01	0.15	3	5 = linear
80	0.15	3	5 = linear

Table S6. Parallel reaction monitoring details. (NCE = normalized collision energy; Transitions represent fragments from each peptide precursor m/z. The summed peak areas from quality-controlled transitions give the peptide's total peak area; only transitions with good peak shape and no coeluting interference from other peptides are used)

Peptide	Protein	Precursor (m/z)	Scan start (min)	Scan end (min)	NCE	Transitions used for peak area quantification
NMEIVSTEPLSK	β1	674.3449	30.8	35.8	27	y10, y9, y8, y7
DQGYELLK	β2	483.2506	27.6	32.6	27	y6, y5, y4
FGIQAQTVATNFEK	β3	777.4016	35.8	41.8	27	y11, y10, y9, y8, y7
GVLIAGDLLASYGSLAR	β4	838.4725	56.1	61	27	y13, y12, y11, y10, y9
IIDEECDDGLK	β4	710.3372	31	37	27	y10, y9, y8, y7
GGVVLAVDSR	β5	486.7773	24.7	28.7	20	y5
GGVVLAVDSR^	β5	491.7814	24.7	28.7	20	y5
ISVAAASK	β5	373.724	12.2	15.7	20	y7, y6, y5, y4
ISVAAASK^	β5	377.7311	12.2	15.7	20	y7, y6, y5, y4
WDLTDEEAYDLGR	β5	791.8546	40.1	45.1	27	y12, y11, y10, y9
DAYSGGIVR	β5	469.2405	20.3	22.3	27	y7, y6, y5
DGSSGGVIR	β6	424.2171	10.6	12.6	27	y7, y6, y5, y4
LLAAEVK^	None (ISTD)	376.2438	19.5	22.5	20	y6, y5, y4, y3
ILSELDQSYLK	V-37B	654.8559	36.5	39.5	27	y9
LQELSELGEQLSK	V-37B	737.3934	38.5	41.5	27	y12, y11, y10, y9, y8, y7
DGVILGADTR	β7	508.7722	25	28.6	27	y7, y6, y5
VVDVEEVSVQR	β7	629.8355	26.5	29.8	27	y10, y9, y8, y7, y6
TITLEVEPSDTIENVK	UBB	894.4673	39.2	42.9	20	y13, y12, y11, y10, y9, y8
TITLEVEPSDTIENVK^	UBB	898.4744	39.2	42.9	20	y13, y12, y11, y10, y9, y8
NQYDSDVTVWSPQGR	α1	876.4028	32	36.4	27	y12, y11, y10, y9, y8
DTLPPEVDLTTK	α1	664.8508	33	38	27	y10, y9, y8, y7, y6
ASNGVVIATENK	α2	601.8224	18	21.6	27	y10, y9, y8, y7, y6
VPESVYLEAER	α3	646.3301	27.4	32	27	y10, y9, y8, y7, y6
LLDEVFASEK	α4	575.8032	36	39.7	27	y9, y8, y7, y6, y5
NEATLEATR	α4	502.754	14	16	27	y8, y6, y5
GVNTFSPEGR	α5	532.262	19.5	22.2	27	y8, y7, y6, y5
QTEANSYLEK	α6	591.7855	17.5	20	27	y9, y8, y7, y6, y5
LTVEDPVTLEYITR	α7	824.9433	46	50	27	y12, y11, y10, y9, y8, y7

DIYTGDSVSINIITK	β1	819.9329	43.2	46.2	27	y13, y12, y11, y10, y9, y8
LAINLPNFK	β2	515.3082	42.9	46.9	27	y8, y7, y6, y5
GENCVSIAADR	β3	596.2748	18.9	20.9	27	y9, y8, y7, y6
YSFSLTTFSPSGK	α2	711.351	44.2	46.2	27	y12, y11, y10, y9, y8, y7
LDILDTAGQEEFSAMR	Ras like-2	898.4302	45.4	49.4	27	y13, y12, y11, y10, y9, y8
AVWQEEAQQLAR	Ras like-2	714.8651	35.6	37.6	27	y10, y9, y8, y7
